# Burnout and staff turnover among certified nursing assistants working in acute care hospitals during the COVID-19 pandemic

**DOI:** 10.1371/journal.pone.0290880

**Published:** 2023-08-30

**Authors:** Rachel L. Snyder, Laura E. A. Barnes, Katelyn A. White, Ronda L. Cochran

**Affiliations:** Division of Healthcare Quality Promotion, Centers for Disease Control and Prevention, Atlanta, Georgia, United States of America; Stamford Health, UNITED STATES

## Abstract

**Introduction:**

Healthcare worker burnout is a growing problem in the United States which affects healthcare workers themselves, as well as the healthcare system as a whole. The goal of this qualitative assessment was to understand factors that may lead to healthcare worker burnout and turnover through focus groups with Certified Nursing Assistants who worked in acute care hospitals during the COVID-19 pandemic.

**Methods:**

Eight focus group discussions lasting approximately 30 minutes each were held remotely from October 2022–January 2023 with current and former Certified Nursing Assistants who worked during the COVID-19 pandemic in acute care hospitals. Participants were recruited through various sources such as social media and outreach through professional organizations. The focus groups utilized open-ended prompts including topics such as challenges experienced during the pandemic, what could have improved their experiences working during the pandemic, and motivations for continuing or leaving their career in healthcare. The focus groups were coded using an immersion-crystallization technique and summarized using NVivo and Microsoft Excel. Participant demographic information was summarized overall and by current work status.

**Results:**

The focus groups included 58 Certified Nursing Assistants; 33 (57%) were current Certified Nursing Assistants and 25 (43%) were Certified Nursing Assistants who no longer work in healthcare. Throughout the focus groups, five convergent themes emerged, including staffing challenges, respect and recognition for Certified Nursing Assistants, the physical and mental toll of the job, facility leadership support, and pay and incentives.

**Conclusions:**

Focus group discussions with Certified Nursing Assistants identified factors at individual and organizational levels that might contribute to burnout and staff turnover in healthcare settings. Suggestions from participants on improving their experiences included ensuring staff know they are valued, being included in conversations with leadership, and improving access to mental health resources.

## Introduction

In May 2022, the Surgeon General of the United States (U.S.) released an advisory announcing the urgent need to address healthcare worker (HCW) burnout and resignation [1]. Burnout is defined as an occupational phenomenon by the 11th Revision of the International Classification of Diseases (ICD-11) and is “conceptualized as resulting from chronic workplace stress that has not been successfully managed. It is characterized by three dimensions: feelings of energy depletion or exhaustion; increased mental distance from one’s job, or feelings of negativism or cynicism related to one’s job; and reduced professional efficacy” [2]. The challenges of the Coronavirus disease 2019 (COVID-19) pandemic have aggravated already high rates of HCW burnout in the U.S., with several studies estimating that around half of HCWs have experienced burnout during the pandemic [3, 4]. Current literature on burnout is largely focused on physicians and nurses, and data including other roles are less common. However, inclusion of other roles in research related to burnout and turnover is important, as over the next several years, it is expected that there will be a considerable shortage of HCWs in low wage positions [5] and in a study of over 20,000 U.S. HCWs, Certified Nursing Assistants (CNA) were among the groups with the highest levels of stress [3].

In addition to affecting individual HCWs themselves, HCW burnout has also been associated with decreases in patient safety, including higher rates of healthcare-associated infections [6]. Additionally, burnout is associated with increased rates of HCW turnover [7, 8], which leads to higher costs for healthcare systems as a whole. An estimated $4.6 billion are spent on burnout-related turnover for physicians alone in the U.S. each year [9]. To build a more resilient healthcare system overall, more research is needed on the causes of HCW burnout and possible interventions to address this urgent issue. The goal of this project was to gain insight on drivers behind HCW burnout and turnover during the COVID-19 pandemic through exploratory focus group discussions with both current and former CNAs.

## Methods

From October 2022 to January 2023, eight virtual qualitative focus group discussions were held over Zoom with CNAs who worked in acute care hospitals during the COVID-19 pandemic. In order to obtain a broader understanding of factors affecting burnout and turnover among CNAs, both current and former CNAs were included in the focus group discussions. Participants were recruited by Rainmakers Strategic Solutions [10] in partnership with Focus Forward, a qualitative recruitment agency through multiple sources, including social media and outreach via professional organizations. Participation was voluntary and participants were compensated for their time. This activity was reviewed by CDC and was conducted consistent with applicable federal law and CDC policy (See e.g., 45 C.F.R. part 46, 21 C.F.R. part 56; 42 U.S.C. §241(d); 5 U.S.C. §552a; 44 U.S.C. §3501 et seq.). Participants provided verbal consent prior to the start of the focus groups. Per determination by the CDC’s National Center for Emerging and Zoonotic Infectious Diseases (NCEZID) Human Subjects Advisor, this qualitative assessment does not meet the definition of research under 45 C.F.R. 46.102(l) and IRB review is not required. NCEZID’s determination holds that the project did not require submission to CDC’s Human Research Protection Office as granting authority is delegated to the CDC Centers, Institutes, and Offices under CDC Policies SSA-2010-01 and SSA-2010-02.

Each focus group ranged from four to 10 participants and lasted approximately 30 minutes, with the discussion guided by a trained moderator [RC] utilizing open-ended prompts. Topics explored included challenges experienced as a CNA during the pandemic, what participants would change about their experience as a CNA during the pandemic, messages they would like to convey to management in their facility, what could have helped them continue to work as a CNA in an acute care hospital and motivations for continuing or leaving their career as a CNA. The focus groups were recorded with participant permission, transcribed and deidentified, and then coded across the discussions using an immersion-crystallization technique [11] as a group by a team of three coders [RS, RC, LB] with experience in qualitative coding. Codes were summarized using NVivo (released in March 2020) [12] and Microsoft Excel and reviewed as a group to ensure reliability. Consensus in assigned codes was reached through group discussion and codes were then categorized into corresponding themes. Participant demographic information obtained by Rainmakers Solutions during the recruitment process was also summarized overall and by current work status. After deidentification, authors did not have access to information which could identify individual participants.

## Results

Of the 58 CNAs who participated in the focus groups, 33 (57%) worked in an acute care hospital during the COVID-19 pandemic and remained employed as hospital-based CNAs, while 25 (43%) were former CNAs who worked in an acute care hospital at some point during the COVID-19 pandemic but were no longer working in a healthcare setting. Of the 58 participants, 54 (93%) were female, and 41% were White, while 40% were Black or African American (Table 1). Twenty-one percent of the participants were of Hispanic, Latino/Latina or Spanish origin. The mean age of the participants was 39 years, and the participants had a mean of 12 years of experience as a CNA. The participants worked varying shifts, with 27 (47%) working primarily day shift and 20 (34%) primarily working night shift.

**Table 1:** Demographics of participating certified nursing assistants working in acute care hospitals during the COVID-19 pandemic.

	Total (N = 58)		Current Certified Nursing Assistant (N = 33)		Former Certified Nursing Assistant (N = 25)	
	N (%)		N (%)		N (%)	
**Gender**						
Female	54	(93%)	31	(94%)	23	(92%)
Male	4	(7%)	2	(6%)	2	(8%)
**Race**						
White	24	(41%)	10	(30%)	14	(56%)
Black or African American	23	(40%)	17	(52%)	6	(24%)
Two or more races	6	(10%)	3	(9%)	3	(12%)
American Indian or Alaskan Native	1	(2%)	0	(0%)	1	(4%)
Prefer Not to Respond	4	(7%)	3	(9%)	1	(4%)
**Hispanic, Latino/Latina, or Spanish Origin?**						
Yes	12	(21%)	6	(18%)	6	(24%)
No	43	(74%)	25	(76%)	18	(72%)
Prefer Not to Respond	3	(5%)	2	(6%)	1	(4%)
**Age**						
<30	13	(22%)	5	(15%)	8	(32%)
30–39	20	(34%)	13	(39%)	7	(28%)
40–49	15	(26%)	12	(36%)	3	(12%)
≥50	10	(17%)	3	(9%)	7	(28%)
**United States Census Region**						
South	26	(45%)	16	(48%)	10	(40%)
West	21	(36%)	12	(36%)	9	(36%)
Northeast	6	(10%)	2	(6%)	4	(16%)
Midwest	5	(9%)	3	(9%)	2	(8%)
**Years of Experience as Certified Nursing Assistant**						
<5 Years	11	(19%)	4	(12%)	7	(28%)
5–9 Years	14	(24%)	8	(24%)	6	(24%)
10–14 Years	15	(26%)	10	(30%)	5	(20%)
≥15 Years	18	(31%)	11	(33%)	7	(28%)
**Shift Most Frequently Worked**						
Day	27	(47%)	18	(55%)	9	(36%)
Night	20	(34%)	10	(30%)	10	(40%)
Other or Multiple	11	(19%)	5	(15%)	6	(24%)
**If Former Certified Nursing Assistant, Current Work Status**						
Working Outside of Healthcare	-		-		12	(48%)
Unemployed	-		-		9	(36%)
Retired	-		-		4	(16%)

Throughout the focus groups, five convergent themes emerged when participants discussed challenges experienced, what they would change about their experience working as a CNA during the pandemic, messages they would like to convey to management in their facility, what could have helped them continue to work as a CNA, and their motivations for continuing or leaving their career as a CNA (Table 2).

**Table 2:** Convergent themes expressed by participating certified nursing assistants working in acute care hospitals during the COVID-19 pandemic.

Theme	Number of Unique Participants Who Expressed Theme		
	Overall (N = 58)	Current Certified Nursing Assistant (N = 33)	Former Certified Nursing Assistant (N = 25)
	N (%)	N (%)	N (%)
Theme 1: Staffing	41 (71%)	22 (67%)	19 (76%)
Theme 2: Respect & Recognition for Certified Nursing Assistants	37 (64%)	21 (64%)	16 (64%)
Theme 3: Physical & Mental Toll of the Job	33 (57%)	15 (45%)	18 (72%)
Theme 4: Support from Facility Leadership, Including Availability of Supplies & Communication	32 (55%)	17 (52%)	15 (60%)
Theme 5: Pay & Incentives	29 (50%)	22 (67%)	7 (28%)

### Theme 1: Staffing

The most convergent theme which emerged was related to staffing, with over 70% of the participants directly mentioning staffing as a problem in their facilities. The CNAs consistently discussed the high patient-to-CNA ratios, which one CNA described as being *“absolutely out of control*. *When you try to let them (leadership) know*, *‘Listen*, *I have a 30 to 40 to 50 person ratio*. *I am the only CNA on the floor*, *I need assistance*. *I don’t feel safe with these numbers*.*’ No one is hearing us*. *It’s going on deaf ears*.*”* Several CNAs also expressed concern that the high patient-to-CNA ratios were affecting their ability to provide quality patient care, and that *“20 or 25 patients for just one CNA*, *that is ridiculous*. *How can you have your patients clean and safe if it is only one CNA for so many patients*?*”*

Several current CNAs described that the staffing ratios during the pandemic led them to consider leaving work in direct patient care, while former CNAs described that they left because severe staffing shortages prevented them from providing proper care to their patients. One former CNA stated, “*Morally*, *I want to do my job and I want to do it right*. *There is no way for me to do it the right way without proper staffing*, *proper Personal Protective Equipment*.*”*

The CNAs also mentioned the effect that the use of HCWs from staffing agencies had on their facilities, including that *“agency staff would jump around and work in your facility today*, *another facility tomorrow*, *so they never knew the patients*. *They never knew the routine*. *That caused so much havoc and so much chaos*.*”*

Some suggestions provided by the CNAs to help address staffing issues in their facilities included redirecting efforts to “*focus on retention instead of sign-on*… *They need to focus on retention and keep the good people that they have*. *Take care of them financially*.*”* Another CNA suggested to “*hire more people and actually train them*, *so that they wouldn’t just leave after their first shift or in the middle of their first shift*. *Better training (is needed)*. *We hired kids straight out of high school who were just doing it for money*. *And they would give them a two-week orientation and expect them to know everything*.*”*

### Theme 2: Respect and recognition for CNAs

The next most convergent theme that emerged throughout the focus group discussions was the feeling that CNAs were not respected or recognized in their facilities. One former CNA described that they “*felt as if there was no support from nursing staff*. *One thing about CNAs is that we are highly intelligent*, *and we are just treated like a second hand sometimes*. *One of the biggest reasons why I left was because of the disrespect*.*”* One CNA described a point during the pandemic where “*the nurses would hand the meds and that was it*. *They would not go back in that room*. *But we (as CNAs) had to constantly bring the trays in the room*, *take the trays out*, *change the patient*, *bring the patient to the bathroom*.” Other CNAs described that problems in the facility “*rolled downhill and so all the problems became my problem (as the CNA) and I just can’t do it anymore*.”

One CNA felt that their facility *“forgot that us CNAs existed*. *We need more awareness that we aren’t just glorified butt wipers*. *We are there to hold your loved one’s hands*, *take care of them*, *comfort them when they are dying or when they are sick*.*”* Another CNA wished they had gotten *“something to show that we worked through the whole COVID pandemic*. *We couldn’t stay at home*. *We had to come to work*. *We had to still take care of people who couldn’t take care of themselves*.*”* The CNAs also specifically mentioned that they received less recognition than other professions in their facilities, including that the facility should *“treat CNA work as equal importance to RN work… I feel like we are just overlooked in general because of license versus degree… Just because we don’t push meds doesn’t mean we aren’t doing the work and don’t deserve the pay*.”

The CNAs also discussed ideas for how facilities could show appreciation, recognition, and respect for CNAs. Several CNAs thought that celebrating CNA Week was one way to specifically show recognition for the work that they do. *“When Nurse’s Week comes*, *they go all out*. *You can do the same thing for CNAs*. *You can appreciate us the same way*, *because we are what makes the business (of healthcare) run*.*”* Another shared that *“I don’t want the gifts*, *but I do want to be celebrated during my week*. *The nurses get celebrated*, *so I would like to get celebrated…*. *I was doing everything for those patients*. *Let’s everybody bring food for our CNAs*. *Celebrate our CNAs*. *Thank them for everything they do for all of us*.*”*

Another way to show recognition that was discussed by the participants was for upper management to directly say thank you to the CNAs. “*Just physically coming down and saying thank you*, *I appreciate what you are doing*. *No*, *I don’t want a cheesy letter that is addressed to everyone (across the hospital)*. *I don’t want pizza for the 12 thousandth time this month*.” One CNA suggested that facilities could also show appreciation by paying for the cost of their parking at work or providing discounts for the cost of scrubs and shoes as *“our shoes are our livelihood*. *Maybe get us discounts for scrubs… Discounts on insoles for our shoes*. *Because it is hard*. *It’s back breaking work*.”

### Theme 3: Physical and mental toll of the job

Over 55% of the participants described the toll that the job took on them, both physically and mentally. One former CNA described that they *“dreaded coming into the hospital for months*. *To the point I had panic attacks right before going into work every time*.*”* Of the 25 participants who were former CNAs, 18 (72%), gave the physical and mental toll as a specific reason for their decision, with one CNA describing that, “*It was causing me to be depressed*. *I watched my clients die without their loved ones…*. *I would just sit in my car and cry*. *I was in a really low place in life working in the hospital*.*”* Another expressed that one of the reasons they left “*was just my back as well*. *After two years of that… I am already going to have chronic back problems because of having to lift so many people for so long… So that was a big struggle*, *a physical toll that it took*.*”*

Several CNAs suggested that improved access to mental health services is needed for CNAs. One CNA described that they **“***worked at two different hospitals during the pandemic and the first one offered some type of counseling but the second one did not offer anything*.” Other CNAs described the need for debriefing after shifts, with one CNA suggesting that “*debriefing and talking about it and explaining how that affected us with other people who were experiencing the same thing would have helped a ton*.” One CNA expressed that **“***there are so many of us that got burnt out*, *that saw so much*. *I ended up being a liaison between patients who were dying and having to do facetime calls with their families*. *That wasn’t anything that I was even trained for or even debriefed on at the end of the shift*. *I would go home and cry*. *These were people saying goodbye to their mothers*, *brothers*, *family members and never being able to see them again*. *And I am trying to hold space for that*. *It was a lot*. *I hope that is something that is addressed in the future*.” One CNA also shared that mental health services are not always offered equally to staff across different roles in facilities, and that “*they offered a mental health service at my work*, *but it didn’t get opened to anyone other than nurses for six months*. *That was tough to think that it wasn’t even being considered for me (as a CNA)*.”

### Theme 4: Support from facility leadership, including availability of supplies and communication

Another convergent theme which emerged was the need for more support from leadership in their facility, both tangible (e.g., availability of supplies) and intangible (e.g., better communication between leadership and CNAs). One CNA described that their facility needed to *“actually have the correct supplies and act like they care if we came in contact with the people [with COVID]*. *Sometimes they wouldn’t even let us know until after you had already been in contact with them*,*”* while another said their facility needed to *“make sure I have all the linen that I need to do my job*. *The Personal Protective Equipment*. *I don’t want to get a mask and have to wear that mask for 7 days*.*”*

Other CNAs expressed that there was *“a lack of support*, *a lack of communication*, *and then also not being included in meetings and planning”* and that *“there is no way in 2023 we should be running low on wipes*, *briefs*, *any of that*. *Simple things that should be ordered based upon the needs of the facility*.*”*

One idea shared by a participant on how their facility supported CNAs was a suggestion box for the nurse’s station, *“You could write things that were negative or things that needed work*, *like constructive criticism*. *Or you could write positive things too*. *And then periodically*, *we would have a little meeting and then go through the box*. *It was kind of a morale booster*. *A lot of the issues we were having*, *some of them collectively we could solve*.*”* Other CNAs suggested that management should listen to CNAs more, including the idea to *“sit down and maybe have a lead CNA who has worked the floor and done the hard work*. *Being open to listening and taking the feedback and maybe changing some things for us*.*”*

### Theme 5: Pay and incentives

Half of the participants discussed pay and incentives for CNAs. Throughout the focus groups, the CNAs consistently described that the pay for their job was too low considering their responsibilities and workload. In the words of one CNA, *“Make sure our pay compensates us*. *Any time McDonalds is paying more than what you are making when you are responsible for a human being*, *that is a major issue*.*”* Other CNAs expressed that *“none of us should have to work two jobs to support ourselves if this is our livelihood*,*”* and that the low pay affected staffing because *“unfortunately*, *for some people looking at the money and the workload*, *it is an undesirable job*.*”*

In addition to higher wages, CNAs also expressed that they were not rewarded with incentives and hazard pay comparable to other HCWs during the pandemic. One CNA expressed that they *“would change the fact that our hazard pay could have been more… we had to do most of the work while the doctors and nurses did not want to come on our unit*. *They paid the nurses ten grand just to pick up a shift and we just got five dollars*. *I don’t think that was fair*.*”* Another CNA described *“a point in time where they put something up on the bulletin board that they were offering incentives for holidays and for coming in*. *And then about three days later*, *they wrote underneath it that it was for ‘RNs ONLY’ and that made it really hard to want to pick up shifts because I (as a CNA) wasn’t getting the kind of money that nurses were being given*, *and my workload was terrible*.*”*

Another CNA described that their hospital did give bonuses for working during the COVID-19 pandemic, however they decided to leave their job once the pandemic bonuses ended and that, *“the tipping point for me*, *was once everything started to settle down in the hospital*, *there were different bonuses that were offered for working during COVID and then all of a sudden it was all taken away*. *And it was like OK*, *the work I am doing is the same*, *but all of a sudden I’m being paid less*.*”*

### Additional themes

In addition to the five most convergent themes described above, of the 33 current CNAs still working in acute care hospitals, 22 (67%) described their passion for helping patients as a motivation for continuing to work as a CNA. In the words of one CNA, “*Even through the pandemic*, *I still love what I do*. *I have seen a lot of loss*, *but I have also seen a lot of people get better*. *I want to leave*, *but my heart is still in it*.*”* However, two of the current CNAs did express that losing their passion has made them consider leaving their position, with one CNA mentioning that she “*even started an online boutique so I can get out of the field*. *As a CNA*, *I used to like to do it because it was rewarding*.” Six of the CNAs also expressed that if they left their position, they were concerned about who else would take care of the patients, with one CNA describing that “I *got to train oncoming CNAs to our unit and I lack confidence in them…there is really not that passion to help people*. *I have stayed because unfortunately I don’t trust the new people being hired to do quality work*.*”*

Another motivation for career decisions that emerged among both current and former CNAs was the desire to advance their education. One former CNA described that being a CNA “*made me want to go further in my career*. *I’m going to start nursing school*, *and it (being a CNA) helped me learn a lot*,*”* while another *“ended up feeling really helpless on a lot of different shifts*. *I felt like I needed to do something more*, *like I needed to have some sort of a wider range of knowledge*. *I actually decided to go to medical school*.*”* Several other participants described that they left their role as a CNA because of concerns about the state of the healthcare system in the U.S., with one CNA describing that they left due to “*disappointment in how America’s healthcare system is run*,” while another “*felt like this is never going to get better*.”

## Discussion

During the COVID-19 pandemic, healthcare systems across the U.S. were faced with a surge of patients requiring care along with shortages of Personal Protective Equipment and staff [13]. Convergent themes that emerged across the focus groups included staffing challenges, a lack of respect and recognition in their facilities, the toll the job takes on CNAs both physically and mentally, a lack of facility leadership support for CNAs, and the need for better pay and incentives for CNAs.

The participants shared their experiences working in facilities with patient-to-staff ratios reported as high as 40 to 1 and the effect these ratios had on their day-to-day experiences as CNAs. The CNAs described that high patient-to-provider ratios were a factor that led them to consider leaving their job, potentially further exacerbating the issue. One CNA described that learning about high patient-to-staff ratios when applying to a job in a new facility would prevent them from wanting to work there. Staffing shortages also have been reported across healthcare facility types and worker roles both before and during the pandemic [14–17], with a qualitative study of nursing home staff also finding that staffing was a main concern for CNAs in long-term care settings [18]. In a survey of over 20,000 physicians and nurses in the U.S. during the COVID-19 pandemic, over half of nurses felt that staffing levels were not adequate to properly care for patients and improved nurse staffing was the intervention respondents felt was most important for improving their well-being [19]. While various studies have focused on the relationship between staffing ratios for Registered Nurses and patient safety [20], evidence to more clearly define staffing ratios for CNAs is very limited, and more research in this area is warranted. Understanding the implications for both patient safety and for HCW well-being should be included as part of further investigations.

The CNAs in this analysis also expressed that the mental and physical toll that being a CNA placed on them contributed to burnout. Across healthcare settings, CNAs provide essential care to patients, including turning, bathing, and feeding. As the role of a CNA often includes manual handling of patients and other tasks which can be physically taxing, the physical exhaustion experienced during their jobs was described as one reason that CNAs considered leaving their positions. The CNAs also described that facilities did not routinely offer mental health support, or that this support was not consistently offered for staff in all roles. Offering mental health support and resources to HCWs across all roles in facilities, especially considering the high rates of anxiety, depression, and Post Traumatic Stress Disorder seen in HCWs during the pandemic [21], might help address this issue. Several participants also described that they became morally uncomfortable with what they perceived as the decreased quality of care they were able to provide to patients due to limited staffing and resources. This sentiment is similar to the moral distress and moral injury that has previously been described in HCWs which stems from limitations outside of their control preventing them from being able to provide the care that they feel their patients need [22, 23]. These concepts should also be considered in addition to burnout when discussing interventions to improve the HCW experience and decrease turnover.

The theme of lack of respect and recognition for CNAs which emerged during the focus groups is concordant with a nationwide study that found that HCWs who felt valued by their facilities experienced lower rates of stress and burnout [3]. The U.S. Surgeon General Advisory also includes ensuring that workers know they are valued as one way that healthcare organizations can work to address burnout [1].

Specific suggestions for improvement at the healthcare facility level provided by the focus group participants included making CNAs feel valued by celebrating CNA week, upper management directly saying thank you to CNAs, improving wages and hazard pay, and giving discounts for essentials such as work shoes, scrubs, and parking. However, frequent pizza parties and impersonal thank you letters from management were spoken of negatively by participants. The participants also thought that debriefing with their peers after traumatic situations may improve their mental health. Several studies have shown the feasibility of debriefing interventions to support HCWs at risk for burnout, and additional research further exploring debriefing programs may be important in developing tools for prevention of HCW burnout in the future [24, 25]. The CNAs also desired to be included in conversations with facility leadership about improvements that could be made in their facilities. As suggested by one participant, having a lead CNA to act as a liaison between CNAs and facility leadership may provide the opportunity for CNA participation in workplace experience improvement. Creating a forum for CNAs to have regular contact with leadership to discuss their work experiences and opportunities for improvement may also serve to facilitate support for CNAs. Previous qualitative discussions with HCWs during the COVID-19 pandemic have also cited the importance of leadership who listen to and take action to address the concerns of frontline personnel [26] and a survey of over 30,000 U.S. HCWs found that favorable leadership scores were associated with lower odds of burnout [27].

Various interventions to address and prevent HCW burnout have been cited in the literature, including individual-level prevention measures (e.g., exercise, self-care training, and improved sleep) and organizational interventions, such as paid time off or inclusion of input from HCWs across various roles when making decisions that impact HCW policy and practice in facilities [1, 28]. Improving both individual and organizational factors is important for preventing burnout and supporting workers who are experiencing burnout.

Our study had several limitations. Participants represented a voluntary convenience sample and all data collected were self-reported. Data were also subject to recall bias and social desirability bias. Participants may not be representative of the overall population of CNAs in the U.S.; and therefore, generalizability of participant perceptions may be limited. Compared to the overall population of CNAs in the U.S., the sample of participants in our focus groups had an overrepresentation of CNAs from the South and West Regions of the U.S. and an underrepresentation of participants who were of Asian or Pacific Islander descent. Despite this, the overall demographics of our participants were similar to the overall population of CNAs in the U.S., with U.S. Census Bureau data estimating that in 2020, 89% of CNAs in the U.S. were Female, while 50% were White, 35% Black or African American, 5% Asian or Pacific Islander, 5% were people of other races, 4% people of more than one race, and 0.9% were American Indian or Alaskan Native [29]. As the CNA workforce largely consists of women and people of color, further research on workplace-associated inequities among CNAs (e.g., unequal access to mental health resources) is important as they may further exacerbate health inequities experienced by women and people of color overall. Additionally, it is also important to note that over 20% of CNAs in the U.S. are foreign-born and further research on inequities experienced by immigrant health workers may also be an important topic to consider, as this population is recognized as having their health and well-being disproportionately impacted by the COVID-19 pandemic [1]. Furthermore, when looking at potential differences between themes expressed by current and former CNAs during the focus groups, caution should be taken in interpretation due to the qualitative nature of these open-ended focus group discussions. While a somewhat higher proportion of former CNAs expressed the theme of the physical and mental toll of their jobs in comparison to current CNAs, and a somewhat lower proportion of former CNAs discussed pay and incentives as compared to current CNAs, these qualitative data should not be used as a basis for statistical comparison between the two groups.

In summary, focus groups with CNAs in acute care hospitals have shown that factors at the individual and organizational levels affect the HCW experience, contribute to burnout and turnover, and should be addressed to ensure a resilient healthcare system. The focus group participants and CNAs nationwide provided essential care to patients throughout the COVID-19 pandemic and, as such, should be included in activities to recognize their contributions and provided equal access to resources for support. Additionally, as literature related to HCW burnout largely focuses on nurses and physicians, more research is needed on factors leading to burnout among workers in other healthcare roles and developing evidence-based interventions to prevent burnout in these groups.
